# GV1001, hTERT Peptide Fragment, Prevents Doxorubicin-Induced Endothelial-to-Mesenchymal Transition in Human Endothelial Cells and Atherosclerosis in Mice

**DOI:** 10.3390/cells14020098

**Published:** 2025-01-10

**Authors:** Wei Chen, Seojin Kim, Sharon Y. Kim, Cheyenne Beheshtian, Naryung Kim, Ki-Hyuk Shin, Reuben H. Kim, Sangjae Kim, No-Hee Park

**Affiliations:** 1 The Shapiro Family Laboratory of Viral Oncology and Aging Research, UCLA School of Dentistry, University of California, 714 Tiverton Ave, Los Angeles, CA 90095, USA; wchen@dentistry.ucla.edu (W.C.); sj25kim@gmail.com (S.K.); skim@dentistry.ucla.edu (S.Y.K.); cbeheshtian@dentistry.ucla.edu (C.B.); naryungkim@dentistry.ucla.edu (N.K.); kshin@dentistry.ucla.edu (K.-H.S.); rkim@dentistry.ucla.edu (R.H.K.); 2 UCLA Jonsson Comprehensive Cancer Center, 10833 Le Conte Ave, Los Angeles, CA 90095, USA; 3 Teloid Inc., 920 Westholme Avenue, Los Angeles, CA 90024, USA; 4 Department of Medicine, David Geffen School of Medicine at UCLA, University of California, 10833 Le Conte Ave, Los Angeles, CA 90095, USA

**Keywords:** GV1001, doxorubicin, endothelial-to-mesenchymal transition, mitochondria, atherosclerosis

## Abstract

Doxorubicin is a highly effective anticancer agent, but its clinical use is restricted by severe side effects, including atherosclerosis and cardiomyopathy. These complications are partly attributed to doxorubicin’s ability to induce endothelial-to-mesenchymal transition (EndMT) in vascular endothelial cells, a critical process in the initiation and progression of atherosclerosis and cardiomyopathy. GV1001, a multifunctional peptide with anti-inflammatory, anti-cancer, antioxidant, and anti-Alzheimer’s properties, has demonstrated inhibition of EndMT. We investigated whether GV1001 could counteract doxorubicin-induced EndMT in endothelial cells and prevent atherosclerosis in a mouse model. The results revealed that GV1001 significantly suppressed EndMT induced by doxorubicin, likely through its protective effects on mitochondria. By mitigating mitochondrial damage, GV1001 reduced the accumulation of mitochondrial and cellular reactive oxygen species (ROS), repressed the activation of nuclear factor kappa B (NF-κB), and reduced the production of proinflammatory cytokines in endothelial cells. Additionally, GV1001 reduced systemic and vascular inflammation, lipid accumulation, and monocyte/macrophage infiltration within arterial walls in mice. In conclusion, GV1001 appears to prevent doxorubicin-induced atherosclerosis by safeguarding vascular endothelial cells from mitochondrial dysfunction, inflammation, and phenotypic changes. These findings suggest the potential of GV1001 as a therapeutic agent to mitigate the long-term cardiovascular side effects associated with doxorubicin treatment in humans.

## 1. Introduction

Doxorubicin, an anthracycline chemotherapeutic agent, is widely used to treat various malignancies, especially refractory and recurrent tumors [1]. However, its clinical application faces significant challenges due to severe side effects, including doxorubicin-induced cardiotoxicity (DIC) and atherosclerosis [2,3]. While the detailed mechanisms underlying DIC remain unclear, mitochondrial dysfunction is considered one of the major contributors, as doxorubicin preferentially accumulates in mitochondria at concentrations up to 100-fold higher than in plasma [4,5]. Despite available interventions, such as β-blockers and angiotensin II inhibitors, no treatment showed long-term efficacy in preventing or alleviating DIC [6].

Studies demonstrated that doxorubicin induced endothelial-to-mesenchymal transition (EndMT) in vascular endothelial cells, a process characterized by the loss of endothelial traits and the gain of mesenchymal features [7]. EndMT disrupts endothelial barrier function and contributes to both DIC and atherosclerosis. This transition is primarily driven by the upregulation of transforming growth factor beta/suppressor of mothers against decapentaplegic (TGF-β/Smad) signaling pathways, proinflammatory cytokines, and transcription factors such as Snail and Twist [8,9]. Oxidative stress and reactive oxygen species (ROS) were implicated as key drivers of doxorubicin-induced EndMT [10,11].

GV1001 comprises 16 amino acids, which are derived from the hTERT 616–626 sequences (Glu-Ala-Arg-Pro-Ala-Leu-Leu-Thr-Ser-Arg-Leu-Arg-Phe-Ile-Pro-Lys) and locates within the reverse transcriptase domain [12,13], exhibits diverse biological functions, including anti-cancer [14], anti-inflammatory [15,16], anti-oxidant [17], and antiviral properties [18]. GV1001 inhibits proinflammatory cytokines [19], protects against ROS [20], and ameliorates atherosclerosis in preclinical models [15]. Notably, GV1001 attenuates vascular inflammation and prevents EndMT in arterial endothelial cells [15,16]; GV1001 was also reported to have a myocardial protective effect against ischemia–reperfusion injury in laboratory animals [19,21], suggesting its potential cardioprotective effect.

Given its multi-functional properties, GV1001 may serve as a novel therapeutic agent to counteract doxorubicin-induced cardiotoxicity and atherosclerosis. To investigate this potential, we examined its effects on doxorubicin-induced EndMT in vitro, elucidated its underlying mechanisms, and assessed its efficacy in mitigating systemic and vascular inflammation, as well as atherosclerosis, in a murine model.

## 2. Materials and Methods

### 2.1. Cell Culture

Human umbilical vein endothelial cells (HUVEC; #C2519A, Lonza, Basel, Switzerland) were cultured in endothelial basal medium-2 (EBM-2) with EGM-2 SingleQuot Kit (#CC-4176, Lonza, Morristown, NJ, USA). The medium was renewed every 2 days. HUVECs were cultured at 37 °C in 5% *v*/*v* of CO_2_ with humidity.

### 2.2. Animals and Animal Welfare

We purchased thirty 8-week-old male *Apolipoprotein E* (*ApoE*)-deficient mice (C57BL/6 background) from Jackson Laboratory (Bar Harbor, ME, USA). All mice were housed on a high-fat diet (HFD) (#D12079B, Research Diets, New Brunswick, NJ, USA) and given free access to food and water as described previously [15,16]. Mice’s health and behavior were monitored three times per week throughout the experiment. As shown in Figure S1, mice were divided into four groups:

Group 1 (*n* = 7): Intraperitoneal (ip) injections of PBS for 9 weeks.

Group 2 (*n* = 7): ip injections of GV1001 (2 mg/kg) for 9 weeks.

Group 3 (*n* = 8): ip injections of doxorubicin (#25316409, MedChemExpress LLC, Monmouth Junction, NJ, USA) (5 mg/kg) for 8 weeks and PBS for 9 weeks.

Group 4 (*n* = 8): ip injections of GV1001 (2 mg/kg) for 9 weeks and doxorubicin (5 mg/kg) for 8 weeks.

Doxorubicin was administered once a week at a dose of 5 mg/kg via intraperitoneal injection, as this dosage has been shown by others to be appropriate for investigating the doxorubicin-induced atherogenesis in mice [22]. PBS or GV1001 was administered three times per week, and doxorubicin administration began one week after the PBS or GV1001 injections, with doses administered once a week. The intraperitoneal (ip) injection of PBS or GV1001 (2 mg/kg of body weight, obtained from GemVax/Kael, Inc., Seongnam-si, Republic of Korea) was started one week before the doxorubicin injection using a volume of 0.1 mL (Figure S1). GV1001 (2 mg/kg) was administered three times a week via intraperitoneal injection, as our findings [15] have demonstrated this dosage to be effective for its anti-inflammatory effects without causing toxicity.

### 2.3. Sample and Tissue Collections

As shown in Figure S1, we sacrificed the mice after the completion of PBS, GV1001, or doxorubicin injections under general anesthesia and collected whole blood, entire arteries for en face analysis, heart, and spleen as previously reported [16]. Whole blood was collected from mice by cardiac puncture under isoflurane anesthesia (#G46D22, VetOne, Boise, ID, USA). The mice were then perfused with 4% paraformaldehyde (#158127, Millipore Sigma, Burlington, MA, USA) in phosphate-buffered saline (PBS) via the left ventricle for 5 min. Following perfusion, the entire aorta, extending to the iliac bifurcation, was exposed, carefully dissected from surrounding tissues, and preserved in RNAlater (#AM7020, Thermo Fisher Scientific, Waltham, MA, USA). Additionally, the spleen was resected, and its tissue was split into two portions. One half was fixed in 4% paraformaldehyde in PBS (pH 7.4), while the other half was stored in RNAlater at −80 °C for analysis of proinflammatory cytokine expression.

### 2.4. Frozen Sectioning

The heart samples were embedded in cryomolds using Tissue-Tek O.C.T. compound (#M71484, Sakura Finetek, Torrence, CA, USA) and stored at −80 °C until cryosectioning. Frozen heart blocks were sectioned at a thickness of 10 μm at −20 °C using a Cryostar NX70 cryostat (Thermo Fisher Scientific, Waltham, MA, USA), following the procedure described previously [23].

### 2.5. Histological and Immunofluorescence (IF) Analysis

For immunofluorescent staining, the fixed or live cells, paraffin sections of spleen, and frozen sections of aortic roots were incubated with primary antibodies, such as CD31 (cluster differentiation 31, #ab28364, Abcam, Cambridge, UK), α-SMA (alpha-smooth muscle actin, #A2547, Millipore Sigma, Burlington, MA, USA), TNF-α (#ab6671, Abcam, Cambridge, UK), p65 (#SC8008, Santa Cruz Biotechnology, Dallas, TX, USA), p-p65 (#CST3036, Cell Signaling, Danvers, MA, USA), Mitosox Red Mitochondrial Superoxide Indicator (#M36008, Invitrogen, Carlsbad, CA, USA), Fluorometric Intracellular ROS probe (#MAK143, Millipore Sigma, Burlington, MA, USA), MitoTracker Red CMXRos (#CST9082, Cell Signaling Technology, Danvers, MA, USA), BioTrackerTM Mitochondrial FerroGreen live cell probe (Mito-FerroGreen) (#SCT262, EMD Millipore Corp., Burlington, MA, USA), BioTracker Mitochondrial Lipid Peroxide Live Cell Ferroptosis Indicator (#Mct261, Millipore Sigma, Burlington, MA, USA), and MOMA-2 (#MCA519G, Bio-Rad, Hercules, CA, USA), followed by fluorometric detection with Alexa Fluor 488- or 546-conjugated secondary antibodies (#A11029 or #A11010, respectively, Thermo Fisher Scientific, Waltham, MA, USA). Sequentially, the sections were mounted on slides with VECTASHIELD TM anti-fade mounting medium with 4′,6-diamidino-2-phenylindole (DAPI) (#H1200, Vector Laboratories, Burlingame, CA, USA). The immunofluorescent images were taken under a confocal fluorescent microscope (#LSM 700, Carl Zeiss, Oberkochen, Germany). All experiments were performed according to the manufacturer’s guidelines [24,25,26,27].

### 2.6. Serum Lipid and Proinflammatory Cytokines Measurement in Mouse Serum

Levels of total cholesterol (TC), triglycerides (TG), high-density lipoprotein (HDL), and non-HDL were measured using enzymatic assay kits in the UCLA Cardiovascular Core Facility as described previously [16,28].

### 2.7. Quantitative Real-Time Polymerase Chain Reaction (RT-qPCR)

Total RNA was extracted from HUVECs and mouse tissue, reverse transcribed, and qPCR was performed as described previously [16]. Total RNA was extracted from mouse aortic and spleen tissues using the RNeasy Micro Kit (#74004, Qiagen, Valencia, CA, USA) and reverse transcribed following these steps: 5 min at 65 °C, 2 min at 25 °C, and 1 h at 45 °C using the SuperScript^®^ III Reverse Transcriptase Synthesis Kit (#18080044, Thermo Fisher Scientific, Waltham, MA, USA). Quantitative PCR (qPCR) was then performed using PowerUp™ SYBR-Green Master Mix (#A25742, Thermo Fisher Scientific, Waltham, MA, USA), according to the manufacturer’s protocol. Glyceraldehyde 3-phosphate dehydrogenase (Gapdh) was used as a loading control. Fold induction was calculated using the comparative ΔCq method and presented as relative transcript levels (2^−ΔΔCq^). The primer sequences used for RT-qPCR are listed in Table S1 (Supplemental Materials).

### 2.8. En Face Analysis

The en face analysis and the examination of lipid deposition were performed with entire length of the aorta-to-iliac bifurcation as previously described [29]. The entire length of the aorta, from the aortic arch to the iliac bifurcation, was exposed along the ventral midline and carefully dissected from the animal under a stereomicroscope (Zeiss, Stemi 305, Oberkochen, Germany). For en face analysis, the aorta was stained with Sudan IV (#198102, Sigma-Aldrich, St. Louis, MO, USA) and pinned flat with the intimal side facing up. Aortic images were captured using a Nikon digital camera (Nikon D7500 DSLR Camera, Tokyo, Japan) and analyzed with ImageJ Software version 1.48 (NIH, accessed on 11 May 2023, Bethesda, MD, USA).

### 2.9. Induction of EndMT

The induction of EndMT from HUVECs, immunofluorescent staining, and image analysis were described in our previous reports [16]. HUVECs were seeded in a chamber well of a 4-well chamber slide (#C6932, Lab-Tek II, Nunc, Rochester, NY, USA). The cells were treated with doxorubicin (0.25 μM) or 10 μg/mL GV1001 in endothelial growth medium for two days. Following treatment, the cells were fixed with ice-cold 100% methanol (#L13255, Thermo Fisher Scientific, Waltham, MA, USA) for 10 min and permeabilized with 0.1% Tween 20 (#P2287, Sigma-Aldrich, St. Louis, MO, USA) for 20 min. The cells were then blocked with 5% normal goat serum at room temperature for 1 h. Primary antibodies (CD31, cluster differentiation 31, #ab28364, Abcam, Cambridge, UK; and α-SMA, alpha-smooth muscle actin, A2547, Burlington, MA, USA) were applied overnight at 4 °C. Fluorescence-tagged secondary antibodies were applied for 1 h at room temperature (Alexa Fluor 546-conjugated secondary antibody (#A11010, Thermo Fisher Scientific, Waltham, MA, USA) and Alexa Fluor 488-conjugated secondary antibody (#A11029, Thermo Fisher Scientific, Waltham, MA, USA). The cells were washed three times with PBS and mounted with VECTASHIELDTM anti-fade mounting medium with DAPI (#H1200, Vector Laboratories, Burlingame, CA, USA). Immunostaining images were captured using a confocal microscope (Carl Zeiss, LSM 700, Oberkochen, Germany).

### 2.10. Western Blotting

Western blotting was performed on whole-cell extracts (WCEs) from cultured HUVECs, as described previously [29]. WCEs were isolated using radioimmunoprecipitation assay (RIPA) lysis buffer (#89901, Thermo Fisher Scientific, Waltham, MA, USA). The WCEs were then fractionated by SDS-PAGE and transferred to an Immobilon^®^-P membrane (#IPVH00010, Millipore, Billerica, MA, USA). The membrane was incubated sequentially with primary and secondary antibodies, followed by exposure to chemiluminescence reagents (#1705061, Bio-Rad, Hercules, CA, USA) for signal detection.

### 2.11. Enzyme-Linked Immunosorbent Assay (ELISA)

The serum levels of Tumor Necrosis Factor-α (TNF-α), Interleukin (IL)-1β, and IL-6 were measured by enzyme-linked immunosorbent assay (ELISA) using commercial kits (#BMS607-2HS, #BMS6002, or #BMS603, respectively, Thermo Fisher Scientific, Waltham, MA, USA), following the manufacturer’s protocols. The color reaction was stopped with Stop solution (#423001, BioLegend, San Diego, CA, USA), and absorbance was measured immediately at 450 nm using a plate reader (Bio-Rad Laboratories, PR4100, Hercules, CA, USA). The standard curve was constructed by plotting the standard concentrations against absorbance values, and cytokine levels were calculated in pg/mL [30].

### 2.12. Cell Migration Assay

For the scratch wound healing assay, HUVECs were seeded onto 12-well plates at a density of 1.5 × 10^5^ cells/mL in starvation medium containing 0.5% fetal bovine serum (FBS, #10082147, Thermo Fisher Scientific, Waltham, MA, USA). The cells were allowed to adhere for 24 h at 37 °C in 5% CO_2_, after which they were pre-treated with 10 μg/mL GV1001 for 3 h. Following treatment, HUVECs were scratched using a sterile 200 μL pipette tip. The cells were then washed with PBS to remove debris. After 48 h of treatment with 50 ng/mL TNF-α (#210-TA, R&D Systems, Minneapolis, MN, USA) or 0.25 μM doxorubicin at 37 °C, the distance between the scratch wounds was observed microscopically. Images were captured using an inverted microscope (Olympus, Tokyo, Japan) [29]. For the migration assay, a transwell system with a polycarbonate filter (8.0 μm pore size, #3422, Corning, Corning, NY, USA) was used. HUVECs were pre-treated with 10 μg/mL GV1001 for 3 h, then transferred to the upper chamber of the transwell in medium with 0.5% FBS. The lower chamber was filled with a culture medium containing 50 ng/mL TNF-α or 0.25 μM doxorubicin. After incubation for 24 h, non-migrated cells in the upper chamber were removed with a cotton swab. Migrated cells on the bottom side of the membrane were fixed, stained with 1% crystal violet (#C6158, Sigma-Aldrich, St. Louis, MO, USA), and photographed [15]. The wound gap areas and the number of migrated cells were analyzed using ImageJ Software version 1.48 (NIH, accessed 11 May 2023, Bethesda, MD, USA).

### 2.13. ATP Detection Assay

HUVECs were seeded into a 6-well plate at a density of 1.5 × 10^5^ cells/well in 2 mL of EGM-2 medium. The cells were treated with GV1001 (10 μg/mL) with or without doxorubicin (0.25 μg/mL) for 48 h. Intracellular ATP content was measured using the ATP Detection Assay Kit (#700410, Cayman Chemical, Ann Arbor), following the manufacturer’s instructions, as previously described [31,32]. Briefly, after washing the cells with pre-chilled PBS, 1 mL of 1x ATP Detection Sample Buffer was added to each well. The cells were homogenized by pipetting the buffer up and down several times, and the cell lysates were then transferred to pre-chilled tubes. The Reaction Mixture was prepared by mixing D-Luciferin and Luciferase with 1x ATP Detection Assay Buffer. One hundred microliters of the freshly prepared Reaction Mixture and 10 μL of cell lysates were added to a white opaque 96-well plate in triplicates. The plate was covered and incubated at room temperature for 20 min. The ATP concentration was determined by measuring luminescence using a Synergy H1 multimode microplate reader (BioTek Instruments, Winooski, VT, USA).

### 2.14. Statistical Analyses

All graphs were created using GraphPad Prism Software, and statistical analyses were calculated using GraphPad Prism 9 (GraphPad Software, Boston, MA, USA). For multiple comparisons, a one-way ANOVA with a Newman–Keuls test was used. A *p*-value of less than 0.05 was considered significant. All in vitro results were confirmed by at least three independent experiments. Error bars represent mean ± SE.

## 3. Results

### 3.1. GV1001 Prevents the Doxorubicin-Induced EndMT and Mobility of HUVECs

**Figure 1:**
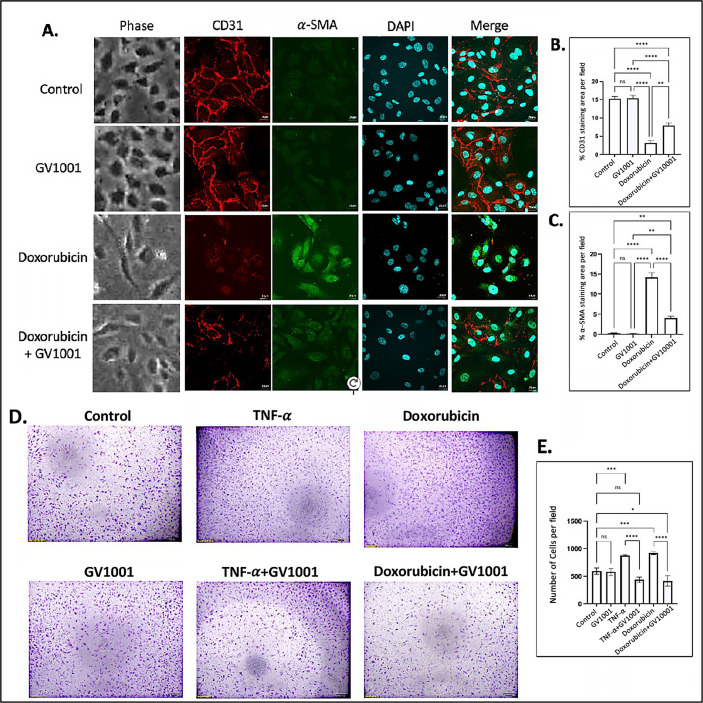
Effect of GV1001 on doxorubicin-induced EndMT and migration of HUVECs. (**A**) Representative immunofluorescent-staining images showing CD31 (red), α-SMA (green), and DAPI (blue) in HUVECs after a 48h exposure to doxorubicin (0.25 μM) and/or GV1001 (10 μg/mL). (**B**,**C**) display the quantification of the fluorescent intensity of CD31 and α-SMA per represented field, analyzed with ImageJ. Statistical significance is indicated as follows: ns (not significantly different), ** *p* < 0.01, **** *p* < 0.0001 with *n* = 5 per group. Scale bar: 20 μm. (**D**) Representative images of migrated HUVECs exposed to GV1001 (10 μg/mL), TNF-α (100 ng/mL), doxorubicin (0.25 μM), or TNF-α+ GV1001 or doxorubicin + GV1001, as assessed with the transwell chamber assay. Images are shown at 40× magnifications. (**E**) Quantification of the number of migrated cells per field. Statistical analysis was performed using one-way ANOVA: ns (not significantly different), * *p* < 0.05, *** *p* < 0.001, **** *p* < 0.0001 with *n* = 5 per group.

Numerous studies have demonstrated that atherosclerosis commences by the disruption of the function of vascular endothelial cells, primarily through EndMT [33]. Previous studies have shown that doxorubicin induces EndMT in endothelial cells, leading to an impaired endothelial barrier function [34,35]. In line with these findings, we observed that doxorubicin induced a morphological shift in endothelial cells toward a mesenchymal phenotype, characterized by downregulation of CD31 and an upregulation of α-SMA in HUVECs. GV1001 effectively suppressed doxorubicin-induced mesenchymal transition, negating the downregulation of CD31 and the upregulation of α-SMA (Figure 1A–C). Western blotting analysis also demonstrated doxorubicin notably reduced CD31 and increased α-SMA levels in HUVECs (Figure S2). GV1001 at 10 ug/mL in culture medium completely reversed this doxorubicin-induced CD31 and α-SMA level changes.

In an in vitro scratch wound healing assay, the migration of HUVECs was significantly increased in response to either TNF-α or doxorubicin. GV1001 almost entirely blocked this migration increase caused by TNF-α or doxorubicin (Figure S3). Similarly, the transwell migration assay demonstrated that both TNF-α and doxorubicin significantly enhanced the HUVEC migration, and GV1001 entirely inhibited the TNF-α- or doxorubicin-induced HUVEC migration (Figure 1D,E). For the migration assay, HUVECs were cultured in the starvation medium (EBM-2 with 0.5% FBS) not only to decrease the potential proliferation of cells by serum but also to maintain HUVECs, reducing the possibility of undernourished adversity. We included TNF-α as a positive control, as it is known to induce EndMT in HUVECs [29]. These results indicate the protective role of GV1001 in shielding endothelial cells from doxorubicin-induced phenotypic changes, suggesting its potential relevance in in vivo atherogenesis.

### 3.2. GV1001 Inhibits the Overexpression of Proinflammatory Cytokines Induced by Doxorubicin in HUVECs

**Figure 2:**
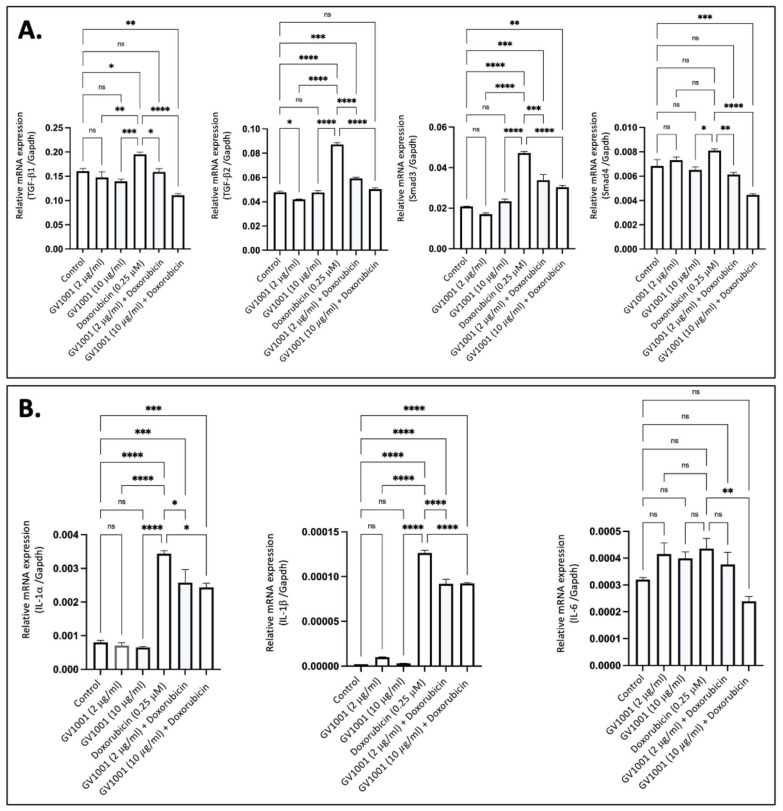
GV1001 reverses the overexpression of proinflammatory cytokines induced by doxorubicin in HUVECs. (**A**) Reversal effect of GV1001 on doxorubicin-induced upregulation of TGF-β1, TGF-β2, Smad3, and Smad4 in HUVECs. Statistical analysis was conducted using one-way ANOVA. (**B**) Reversal effect of GV1001 on doxorubicin-induced upregulation of IL-1α, IL-1β, and IL-6 expression in HUVECs. Statistical analysis was conducted using the one-way ANOVA. Significance levels are indicated as follows: ns = not significantly different, * *p* < 0.05, ** *p* < 0.01, *** *p* < 0.001, **** *p* < 0.0001. Results are presented as mean ± SEM. All experiments were performed in triplicate.

Proinflammatory cytokines, including TGF-β, IL-6, IL-1β, and TNF-α, are known to induce EndMT in endothelial cells [36]. To determine if doxorubicin-induced EndMT is associated with the overexpression of these proinflammatory cytokines, we exposed HUVECs to 0.25 µM doxorubicin and measured cytokine expression levels using RT-qPCR. As shown in Figure 2A, exposure of cells to 0.25 μM doxorubicin significantly increased the expression of TGF-β and its downstream target gene, *Smad3*, in HUVECs; this effect was completely inhibited by either 2 µM or 10 µM of GV1001. Additionally, doxorubicin significantly enhanced the expression of IL-1α, IL-1β, and IL-6 in HUVECs, and GV1001 markedly reduced the doxorubicin-induced overexpression of these cytokines (Figure 2B). These findings suggest that the protective effect of GV1001 against doxorubicin-induced EndMT may be linked to its suppression of proinflammatory cytokine overexpression in HUVECs, as these proinflammatory cytokines induce EndMT in HUVECs [15,37].

### 3.3. GV1001 Inhibits the Doxorubicin-Induced Nuclear Translocation of NF-κB in HUVECs

**Figure 3:**
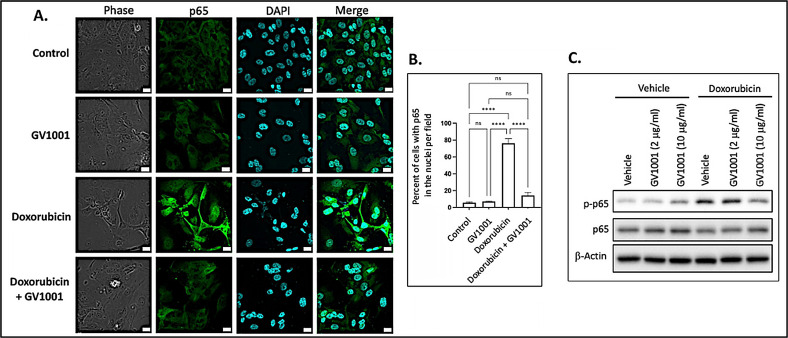
(**A**) Representative immunofluorescent staining images of NF-κB p65 (green) in HUVECs after 48 h exposure of cells to doxorubicin (0.25 μM), GV1001 (10 ug/mL), or doxorubicin + GV1001 (10 μg/mL). (**B**) Percentage of the cells with p65 in the nuclei per field using ImageJ analysis. ns: not significantly different, **** *p* < 0.001. Scale bar: 20 μm. (**C**) Western blotting of phosphorylated p65 (p-p65) and p65 in cells exposed to vehicle only, GV1001 (2 or 10 μg/mL), doxorubicin (0.25 μM), or in a combination of GV1001 with doxorubicin. All experiments were performed in triplicate.

Since activation of NF-κB induces the expression of proinflammatory cytokines [38], we examined whether doxorubicin activates NF-κB by assessing the nuclear translocation of the p50/p65 heterodimer through immunofluorescent staining of p65. As shown in Figure 3A,B, doxorubicin significantly enhanced the immunostained p65 in the nucleus, indicating that doxorubicin activated NF-κB in HUVECs. Notably, GV1001 almost completely inhibited this nuclear translocation of p65, a subunit of NF-κB. We also assessed the effect of doxorubicin on p65 phosphorylation, as phosphorylation of the p65 subunit is crucial for the nuclear translocation of the NF-κB p50/p65 complex to initiate downstream gene transcription [39]. As shown in Figure 3C, doxorubicin markedly increased p65 phosphorylation, while GV1001 inhibited doxorubicin-induced p65 phosphorylation in a dose-dependent manner in HUVECs. These findings suggest that GV1001 suppresses doxorubicin-induced NF-κB activation by inhibiting phosphorylation of p65.

### 3.4. Doxorubicin Induced the Accumulation of Cytoplasmic and Mitochondrial Reactive Oxygen Species (ROS)

**Figure 4:**
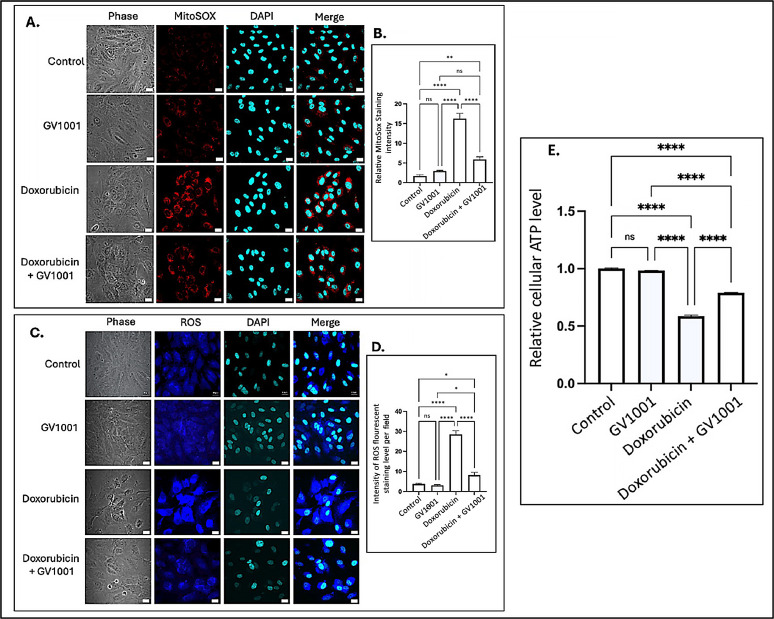
(**A**,**C**) Representative immunofluorescent staining images showing mitochondrial ROS (red) and cytoplasmic ROS (blue) in cells after 48 h exposure to doxorubicin (0.25 μM), GV1001 (10 μg/mL), or a combination of doxorubicin and GV1001 (10 μg/mL). (**B**,**D**) ROS staining intensity was quantified using ImageJ Software. (**E**) Relative cellular ATP levels. Statistical significance: ns = not significant, * *p* < 0.05, ** *p* < 0.01, **** *p* < 0.0001. Scale bar: 20 μm. All experiments were conducted in triplicate.

Intracellular ROS are known to induce inflammation, partly through the activation of NF-κB pathways [40]. Often generated as a byproduct of oxidative phosphorylation, intracellular ROS are primarily produced in mitochondria [41], where electron leakage from the electron transport chain contributes to their production [42]. To investigate the mechanisms by which GV1001 inhibits doxorubicin-induced NF-κB activation, we examined the effects of doxorubicin and the combined GV1001 and doxorubicin on the cellular and mitochondrial ROS levels, as well as cellular ATP levels in HUVECs, using immunofluorescent staining analysis and an ATP detection assay kit as described by others [31,32]. As shown in Figure 4A–D, doxorubicin significantly increased both mitochondrial and intracellular ROS levels. However, GV1001 notably prevented the doxorubicin-induced accumulation of ROS in the cytoplasm and mitochondria. Moreover, cellular ATP level was also diminished by half by doxorubicin, which was almost completely reversed by GV1001, as shown in Figure 4E. These findings indicate that GV1001 effectively mitigates doxorubicin-induced oxidative stress by preventing ROS accumulation in the cytoplasm and mitochondria and restoring cellular ATP levels near to normal.

### 3.5. Doxorubicin Altered the Mitochondrial Structure, and GV1001 Prevented Such Structural Damages

**Figure 5:**
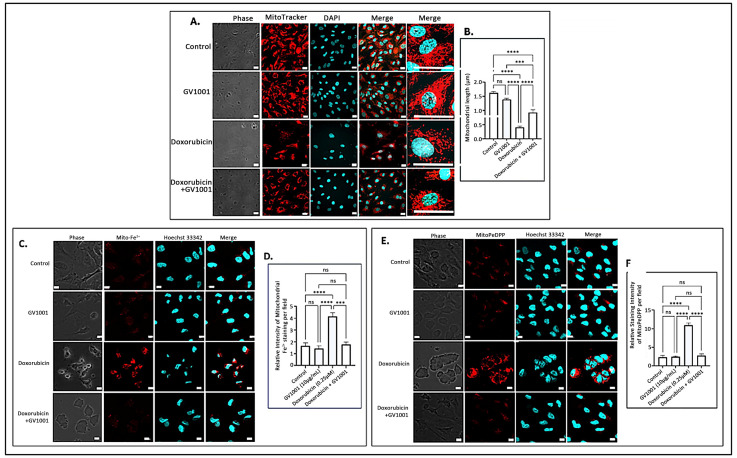
(**A**) Representative immunofluorescent-staining images of mitochondria (red) in fixed HUVECs after 48 h exposure to doxorubicin (0.25 μM), GV1001 (10 μg/mL), or a combination of doxorubicin and GV1001 (10 μg/mL). (**B**) Mitochondrial length measured using ImageJ analysis. Statistical significance: ns, not significant; *** *p* < 0.001; and **** *p* < 0.0001. Scale bar: 20 μm. (**C**,**E**) Representative immunofluorescent staining images of mitochondrial ferrous iron (Mito-Fe^2+^), peroxidated lipid (MitoPeDPP staining), and nucleus (stained with Hoechst nucleic acid staining: Hoechst 33342) of non-fixed HUVECs. (**D**,**F**) The levels of ferrous iron and peroxidated lipid were measured using ImageJ analysis. Statistical significance: ns, not significant; *** *p* < 0.001; and **** *p* < 0.0001. Scale bar: 10 μm. All experiments were performed in triplicate.

The accumulation of mitochondrial ROS leads to significant structural alterations of mitochondria, along with the buildup of mitochondrial ferrous ions [34] and peroxidized lipids [43]. To examine the impact of doxorubicin-induced mitochondrial ROS accumulation, we investigated the effects of doxorubicin on mitochondrial morphology, ferrous iron levels, and lipid peroxidation in HUVECs utilizing immunofluorescence staining. As illustrated in Figure 5A,B, a 48 h doxorubicin treatment substantially shortened mitochondrial length, producing a rounded or short stick-like morphology, which was indicative of fragmented mitochondria. However, GV1001 effectively mitigated these structural changes induced by doxorubicin. Additionally, doxorubicin significantly increased mitochondrial levels of ferrous iron (Figure 5C,D) and peroxidized lipids (Figure 5E,F). Notably, GV1001 entirely prevented these alterations.

### 3.6. GV1001 Inhibits the Doxorubicin-Induced Systemic and Vascular Inflammation in ApoE-Deficient Mice

**Figure 6:**
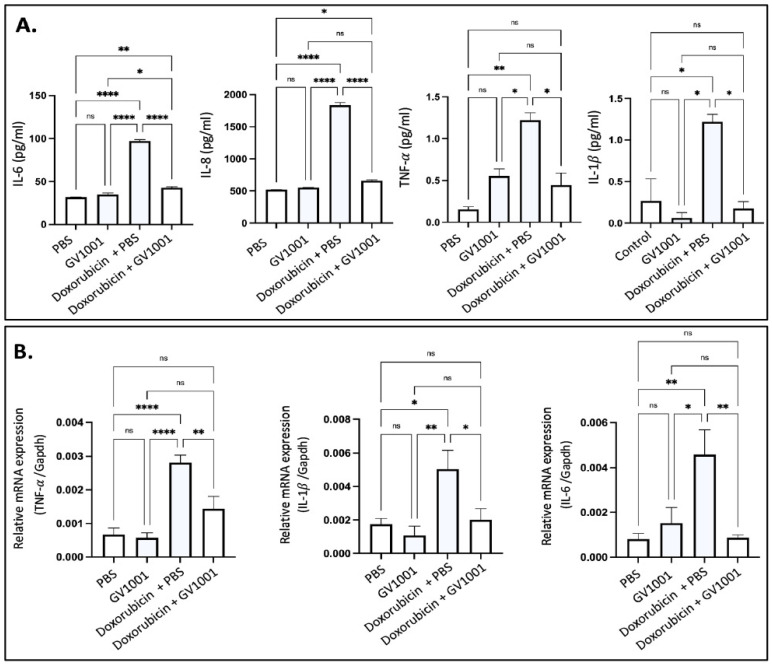
(**A**) Reversing effect of GV1001 on doxorubicin-induced increases in serum levels of proinflammatory cytokines in *ApoE*-deficient mice. Cytokine levels were measured using an ELISA, as described in the Methods section. Statistical analysis was conducted using one-way ANOVA. Symbols denote significance levels: ns, not significantly different; * *p* < 0.05; ** *p* < 0.01, **** *p* < 0.0001. (**B**) Inhibitory effect of GV1001 on doxorubicin-induced increases in the expression of TNF-α, IL-1β, and IL-6 in the arterial tissue. Gene expression was measured via RT-qPCR, with Gapdh serving as a loading control. Statistical analysis was conducted using one-way ANOVA, with significance indicated as follows: ns = not significantly different, * *p* < 0.05, ** *p* < 0.01, **** *p* < 0.0001. Results are presented as means ± SEM, based on 7-8 samples.

Since hyperlipidemia is necessary for the development of atherogenesis, we adopted *ApoE*-deficient mice for the present study, since wild-type mice are not hyperlipidemic and do not develop atherosclerosis [15,16,30]. As shown in Figure S4, doxorubicin suppressed body weight gain in *ApoE*-deficient mice fed a high-fat diet (HFD), with the effect becoming evident two weeks after the initiation of doxorubicin administration. This anti-weight gain effect was not mitigated by GV1001. Doxorubicin is known to induce systemic inflammation [44]. In this study, we evaluated its effects by measuring serum levels of proinflammatory cytokines, including IL-6, IL-8, TNF-α, and IL-1β, in *ApoE*-deficient mice. Doxorubicin treatment significantly elevated all cytokine levels in serum. However, GV1001 effectively prevented these increases (Figure 6A), demonstrating its ability to mitigate doxorubicin-induced systemic inflammation. Since the spleen acts as a principal source of proinflammatory cytokines during systemic inflammation [45], we explored the levels of p-p65 and TNF-α in spleen of mice administrated with doxorubicin and/or GV1001. Interestingly, doxorubicin notably raised p-p65 levels in the nucleus and TNF-α expression in the splenic cells, further confirming spleen’s prominent inflammation effects when stimulated by doxorubicin. GV1001 also reversed these increases, as shown in Figure S5.

Vascular inflammation is a well-known contributor to atherosclerosis [15,16], and doxorubicin was reported to induce such inflammation [46]. To assess the protective effects of GV1001, we determined the expression level of proinflammatory cytokines (TNF-α, IL-1β, and IL-6) in arterial tissues. As shown in Figure 6B, doxorubicin significantly elevated the expression of these cytokines, but GV1001 completely suppressed this increase. These results indicate that GV1001 effectively prevents doxorubicin-induced vascular inflammation in *ApoE*-deficient mice.

### 3.7. Doxorubicin Enhanced the Lipid Deposition in the Arterial Wall, and GV1001 Ameliorated Such Increase

**Figure 7:**
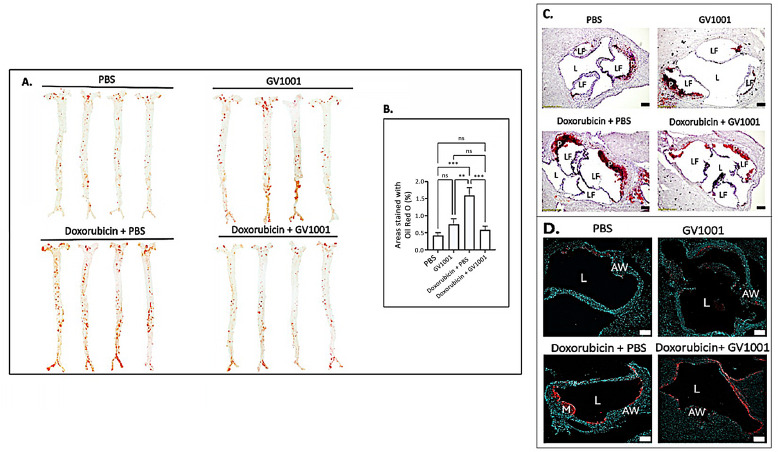
GV1001 inhibited the doxorubicin-induced arterial plaque formation: Inhibition of lipid deposition and macrophage/monocyte infiltration into the arterial wall. (**A**) Representative photographs of mouse arteries from the en face preparation after staining with Oil Red O (8–10 mice per group). (**B**) Quantification of areas of artery stained by Oil Red O using ImageJ analysis. ns, not significantly different; ** *p* < 0.01, *** *p* < 0.001. (**C**) Representative Oil Red O staining of aortic root from the mice. Red color: Lipid; L: Lumen; LF: Leaflet; P: Plaque; (**D**) Representative images of immunofluorescent staining with MOMA-2 (red) in the aortic root of the mice. Nuclei were stained with DAPI (blue). L: Lumen; AW: Arterial Wall; LF: Leaflet; M: Macrophages/Monocytes. Scale bar: 100 μm.

Since vascular inflammation, along with EndMT, is a primary driver of atherosclerosis development [47], we investigated whether the doxorubicin administration enhanced EndMT in arterial endothelial cells, along with possible counteractive effect of GV1001, in the aortic root of mice (Figure S6). Shown in Figure 7 is the impact of doxorubicin and/or GV1001 on lipid deposition and infiltration of macrophages and monocytes in the arterial wall, as previously described [15]. En face analysis revealed minimal lipid deposition in the arterial walls of control mice fed a high-fat diet (HFD) for nine weeks. Systemic administration of GV1001 (2.0 mg/kg) did not alter lipid deposition in these mice. In contrast, treatment with doxorubicin significantly increased lipid deposition in the arterial wall, exceeding three times the levels observed in control mice. Markedly, this increase was completely prevented by GV1001 co-administration (Figure 7A,B). Similarly, the substantial lipid accumulation in the aortic roots caused by doxorubicin was effectively mitigated by GV1001 (Figure 7C). Additionally, doxorubicin treatment resulted in a pronounced increase in macrophage and monocyte accumulation within arterial wall, which was notably reduced when GV1001 was co-administered (Figure 7D).

### 3.8. GV1001 Did Not Impact Doxorubicin-Elicited Changes in Serum Cholesterol Levels

Doxorubicin administration significantly reduced total cholesterol (TC), high-density lipoprotein (HDL), and non-HDL levels in serum, while triglyceride (TG) levels remained unchanged compared to the control group (Figure S7). The reduction in TC levels may be attributed to the downregulation of 3-hydroxy-3-methylglutaryl coenzyme A (HMG-CoA) reductase expression induced by doxorubicin [48]. We also observed that GV1001 did not affect the doxorubicin-induced alterations in cholesterol profiles. Therefore, the anti-lipid deposition effects of GV1001 in mice treated with doxorubicin are likely unrelated to the lipid profile changes caused by doxorubicin.

## 4. Discussion

Doxorubicin, an anthracycline-based chemotherapy agent, is widely used to treat various malignancies, including head and neck cancers, lymphoma, leukemia, bladder cancer, etc. [49]. However, doxorubicin usage is frequently associated with severe long-term side effects, such as atherosclerosis and cardiomyopathy, which can progress to heart failure [50,51]. The atherosclerosis and cardiomyopathy could be partly initiated with the phenotypic alterations of endothelial cells by doxorubicin. Currently, no effective strategies are available to prevent or mitigate these adverse effects.

Our study demonstrated that GV1001 effectively inhibits doxorubicin-induced phenotypic changes in endothelial cells, e.g., EndMT, in human endothelial cells. This effect may result from GV1001’s suppression of doxorubicin-induced overexpression of proinflammatory cytokines. Mechanistically, our findings suggest that GV1001 inhibits NF-κB activation, a critical regulator of proinflammatory cytokine transcription, by inhibiting the phosphorylation p65 subunit and nuclear translocation of activated NF-κB.

As previous studies have indicated that there are correlations between NF-κB activation, mitochondrial dysfunction, and the accumulation of ROS in mitochondria [52,53], we further investigated the effects of doxorubicin on mitochondrial ROS levels and structures. Our findings revealed that doxorubicin induces mitochondrial ROS accumulation, leading to structural abnormalities, lipid peroxidation, ferrous iron accumulation, and impaired ATP production. Remarkably, GV1001 abrogated these mitochondrial impairments caused by doxorubicin. While the exact mechanisms by which GV1001 protects mitochondria remain unclear, our results suggest that GV1001 preserves mitochondrial function, which, in turn, reduces proinflammatory cytokine expression and prevents EndMT in endothelial cells. Mitochondrial dysfunction is implicated in the development of conditions such as atherosclerosis and Alzheimer’s disease [54,55]. Therefore, further studies are warranted to elucidate the mechanisms underlying GV1001’s protective effects against mitochondrial damage.

NF-κB plays a key role in the transactivation of proinflammatory cytokines, including TNF-⍺ [56]. NF-κB activation can also be triggered by TNF-⍺ binding to its receptors [57], potentially leading to systemic inflammation through the nuclear translocation of activated NF-κB and subsequent upregulation of proinflammatory cytokine genes. In our study, mice administered with doxorubicin exhibited elevated TNF-⍺ expression in the spleen and more accumulation of phosphorylated p65 in the splenic cell nucleus, indicating systemic inflammation elicited by doxorubicin may be associated with activation of NF-κB, and subsequently abnormal high expression of TNF-⍺. However, further studies are needed to clarify the relationship between doxorubicin exposure and NF-κB activation in splenic cells, as well as uncovering the mechanisms by which GV1001 inhibits NF-κB activation in the spleen.

Atherosclerosis is driven by chronic systemic and vascular inflammation, characterized by the formation of plaques in the vascular intima that consist of lipids, macrophages, foam cells, and tissue debris [47]. Additionally, EndMT of aortic endothelial cells plays a significant role in the initiation and progression of atherosclerosis by facilitating the accumulation of lipids, monocytes, macrophages, and other leukocytes in the arterial intima [29,58]. Using *ApoE*-deficient mice, we also showed that doxorubicin significantly induced expression and serum levels of proinflammatory cytokines and that GV1001 reversed these adversary effects of doxorubicin. More importantly, GV1001 allowed for preventing lipid deposition in the arterial wall and atherosclerosis development, suggesting that GV1001 may potentially be used as a therapeutic agent for doxorubicin-induced atherosclerosis.

While our study is the first to demonstrate GV1001’s ability to prevent doxorubicin-induced atherosclerosis in mice, its effects should be further investigated in tumor-bearing mice to better understand its therapeutic potential in cancer settings. However, since hyperlipidemia is a prerequisite for atherosclerosis development [24], we had to use hyperlipidemic mice in this study. Nonetheless, this approach presents a potential limitation, as hyperlipidemic conditions, such as those in *ApoE*-deficient or other hyperlipidemic mouse models, restrict tumor development. Future studies should aim to address these challenges by optimizing models that allow concurrent evaluation of tumor progression and atherosclerosis. With these limitations, care should be taken when interpreting the results obtained in this study.

## 5. Conclusions

Although doxorubicin is an effective anticancer agent, its use is limited due to severe side effects, such as cardiomyopathy and atherosclerosis. As GV1001 was reported to inhibit the development and progression of atherogenesis under severe inflammation, we investigated the effect of GV1001 on the development of EndMT and atherosclerosis caused by doxorubicin. As expected, doxorubicin induced mitochondrial dysfunction, activation of NF-κB, and upregulation of proinflammatory cytokines (e.g., IL-6, IL-1β, and TNF-α) in HUVECs, triggering EndMT, a crucial process in the initiation and progression of atherosclerosis. GV1001 effectively mitigated doxorubicin-induced mitochondrial damage, inflammatory responses, and EndMT in vitro. In *ApoE*-deficient mice, systemic administration of doxorubicin resulted in widespread systemic and vascular inflammation. Remarkably, concurrent administration of GV1001 with doxorubicin counteracted doxorubicin-induced inflammation, both systemically and within the vasculature, thereby attenuating the progression of atherosclerosis in these mice. Specifically, GV1001 reduced doxorubicin-induced EndMT, lipid deposition, and macrophage infiltration in the arterial wall. Further investigations, including clinical trials, are essential to confirm the therapeutic potential of GV1001 in mitigating atherosclerosis induced by doxorubicin chemotherapy in cancer patients.

## Data Availability

Data are available upon request to the corresponding author.

## Supplementary Materials

The following supporting information can be downloaded at: https://www.mdpi.com/article/10.3390/cells14020098/s1, Figure S1. In vivo experimental design for assessing the effect of GV1001 on doxorubicin-induced atherosclerosis; Figure S2. Protein levels of CD31 and α-SMA in HUVECs exposed to doxorubicin alone or together with GV1001; Figure S3. GV1001 alleviated Doxorubicin-induced migration of endothelial cells in the scratch wounding healing assay; Figure S4. GV1001 did not counteract the inhibitory effect of Doxorubicin on body weight gain in *ApoE*-deficient mice on a high-fat diet (HFD); Figure S5. GV1001 attenuated doxorubicin-induced TNF-α expression and p-p65 nuclear translocation in splenic cells of *ApoE*-deficient mice; Figure S6. Effect of GV1001 on doxorubicin-induced EndMT in vascular endothelial cells of the aortic roots. Figure S7. GV1001 did not modify the doxorubicin-induced alterations in serum cholesterol profiles of ApoE-deficient mice; Table S1. Sequences of the primers for quantitative reverse transcription–polymerase chain reaction (RT-qPCR).
