# Supplementary material for: GV1001, hTERT Peptide Fragment, Prevents Doxorubicin-Induced Endothelial-to-Mesenchymal Transition in Human Endothelial Cells and Atherosclerosis in Mice
Source: Cells. 2025 Jan 10;14(2):98. doi: 10.3390/cells14020098 (PMC11763685; doi:10.3390/cells14020098)
Supplement: Supplementary file 1 [file cells-14-00098-s001.zip › cells-3392814-supplementary.pdf]

GV1001, hTERT Peptide Fragment, Prevents Doxorubicin-induced Endothelial-to-Mesenchymal Transition in Human Endothelial Cells and Atherosclerosis in Mice

Supplemental Materials

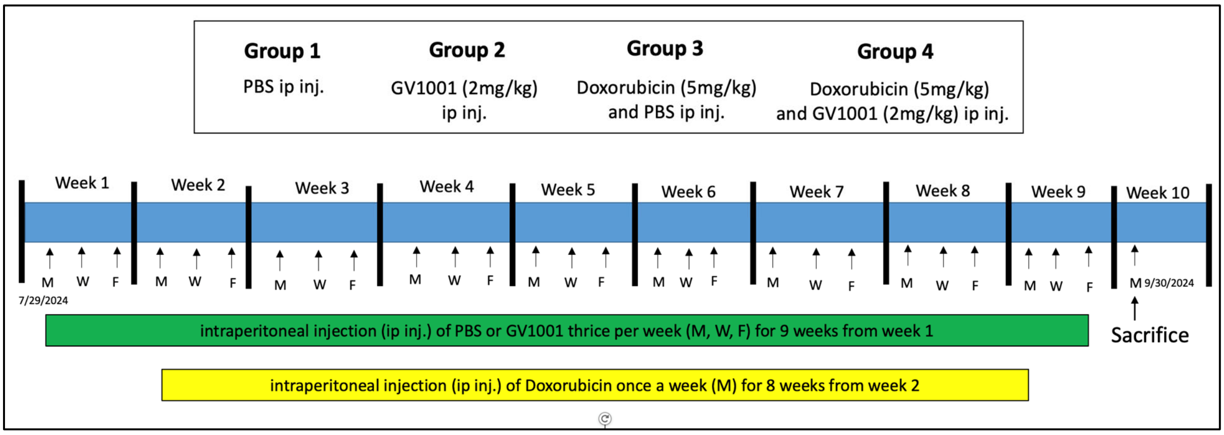

Figure S1. In vivo experimental design for assessing the effect of GV1001 on doxorubicin-induced atherosclerosis. *ApoE*-deficient mice were divided into four groups for the study: (1) Control Group: Mice received intraperitoneal (ip) injections of PBS three times per week for 9 weeks; (2) GV1001 Group: Mice received GV1001 (2.0 mg/kg) ip injections three times per week for 9 weeks; (3) Doxorubicin Group: Mice received PBS ip injections three times per week for 9 weeks, along with doxorubicin ip injections once per week for 8 weeks; and (4) GV1001 + Doxorubicin Group**:** Mice received GV1001 (2.0 mg/kg) ip injections three times per week for 9 weeks, along with doxorubicin ip injections once per week for 8 weeks. M: Monday, W: Wednesday, F: Friday.

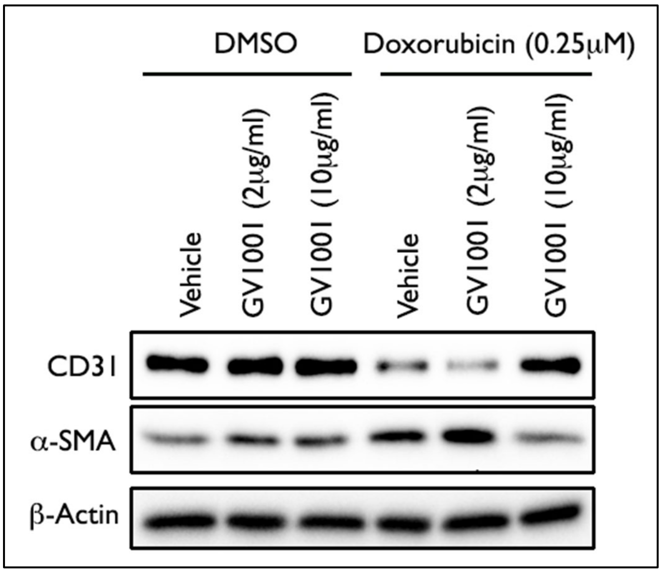

Figure S2. Protein levels of CD31 and α-SMA in HUVECs exposed to doxorubicin alone or together with GV1001. Representative images of western blotting of CD31 and α-SMA in HUVECs exposed to vehicle only, GV1001 (2 or 10 μg/ml), doxorubicin (0.25 μM), or in a combination of GV1001 with doxorubicin. All experiments were performed in triplicate.

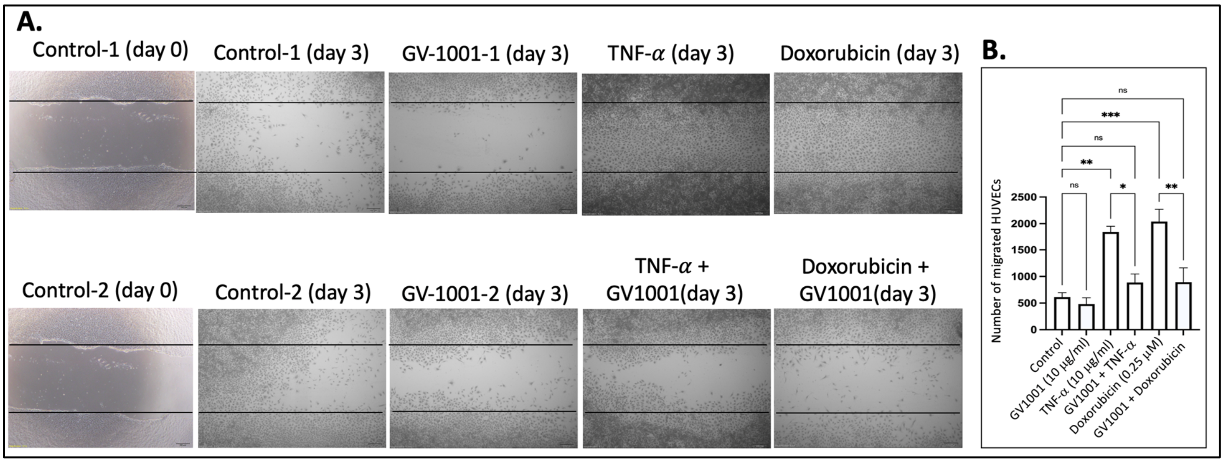

Figure S3. GV1001 alleviated Doxorubicin-induced migration of endothelial cells in the scratch wounding healing assay. **A.** Representative images of migrated HUVECs treated with GV1001 (10 μg/mL), TNF-*α* (100 ng/mL), doxorubicin (0.25 μM), TNF-*α* + GV1001, or doxorubicin + GV1001 for two days after creating a scratch on the culture dishes. Images were captured at 40× magnification. **B.** Quantification of migrated cells per field. Statistical analysis was conducted using a one-way ANOVA, with significance denoted as follows: ns, not significantly different; * $P< 0.05$; ** $P< 0.01$; and *** $P< 0.001$ ($n = 5$ per group).

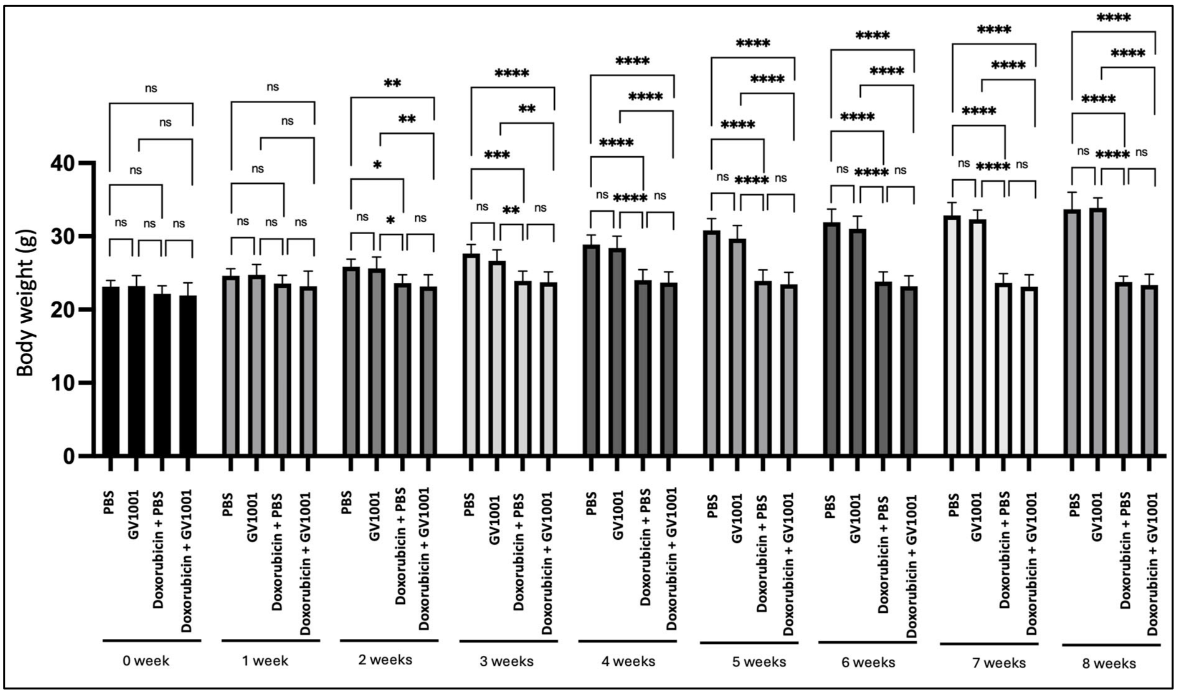

Figure S4. GV1001 did not counteract the inhibitory effect of Doxorubicin on body weight gain in *ApoE*-deficient mice on a high-fat diet (HFD). Statistical analysis was conducted using one-way ANOVA, with significance denoted as follows: ns, not significantly different; * P< 0.05; ** P< 0.01; and **** P< 0.0001 (n = 7 or 8 mice per group).

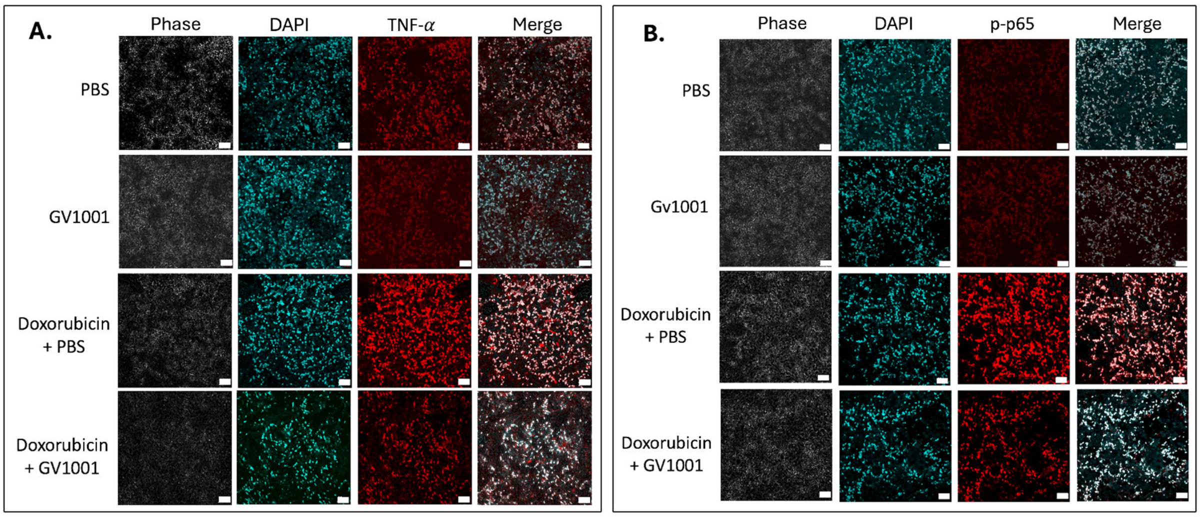

Figure S5. GV1001 attenuated doxorubicin-induced TNF-α expression and p-p65 nuclear translocation in splenic cells of *ApoE*-deficient mice. **A.** Representative immunofluorescent-staining images of splenic TNF-α in *ApoE*-deficient mice. Scale bar: 20 μm. **B.** Representative immunofluorescent staining images of splenic p-p65 in *ApoE*-deficient mice. Scale bar:20 μm

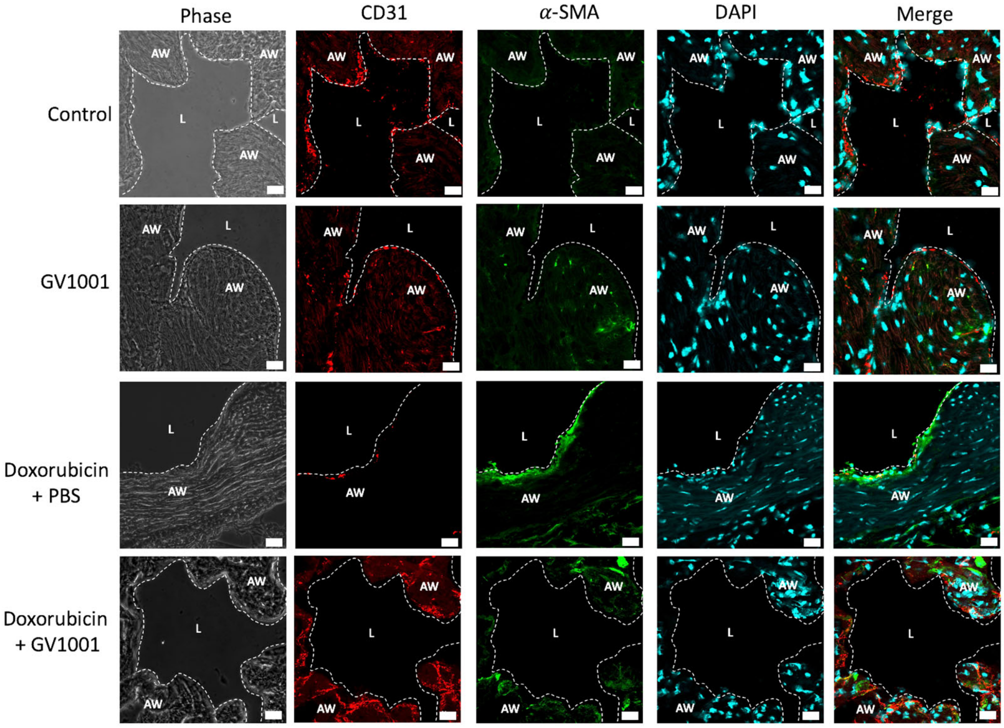

Figure S6. Effect of GV1001 on doxorubicin-induced EndMT in vascular endothelial cells of the aortic roots. Representative immunofluorescent-staining images depict CD31 (red), α-SMA (green), and DAPI (blue) in aortic tissue from mice treated with PBS (Control), GV1001, doxorubicin + PBS, or doxorubicin + GV1001. L: Lumen and AW: arterial wall. Scale bar: 20 μm.

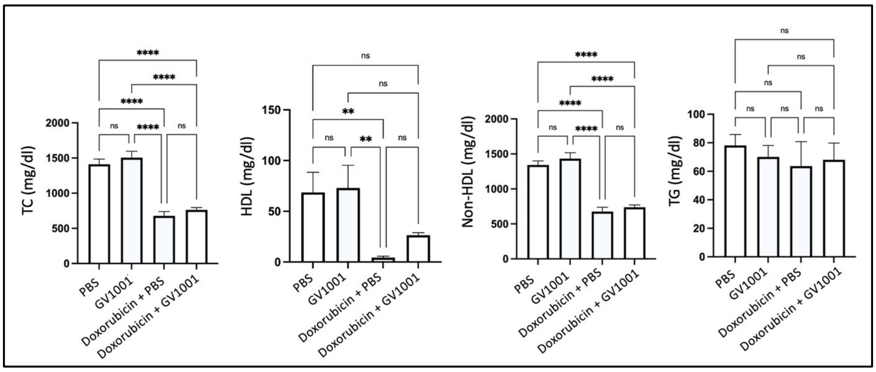

Figure S7. GV1001 did not modify the doxorubicin-induced alterations in serum cholesterol profiles of *ApoE*-deficient mice. Doxorubicin significantly decreased total cholesterol (TC), high-density lipoprotein (HDL), and non-HDL levels, while triglyceride (TG) levels remained unchanged. GV1001 had no effect on these doxorubicin-induced lipid profile changes in mice. Statistical analysis was conducted using one-way ANOVA, with significance levels denoted as: ns, not significantly different; ** P< 0.01; and **** P< 0.0001.

Table S1. Sequences of the primers for quantitative reverse transcription–polymerase chain reaction (RT-qPCR).

Genes	Forward primer 5′-3′	Reverse primer 5′-3′
mIL-1β	CACAGCAGCACATCAACβAAG	GTGCTCATGTCCTCATCCTG
mTNF-α	TCAGGTTGCCTCTGTCTCAG	GCTCTGTGAGGAAGGCTGTG
mIL-6	TGGGACTGATGCTGGTGACA	GCCTCCGACTTGTGAAGTGGT
mGapdh	AGCTTGTCATCAACGGGAAG	TTTGATGTTAGTGGGGTCTCG
hTGF-β1	TACCTGAACCCGTGTTGCTCTC	GTTGCTGAGGTATCGCCAGGAA
hTGF-β2	AAGAAGCGTGCTTTGGATGCGG	ATGCTCCAGCACAGAAGTTGGC
hSmad3	TGAGGCTGTCTACCAGTTGACC	GTGAGGACCTTGTCAAGCCACT
hSmad4	CTACCAGCACTGCCAACTTTCC	CCTGATGCTATCTGCAACAGTCC
hIL-1 α	TGTGACTGCCCAAGATGAAG	CGTGAGTTTCCCAGAAGAAGAG
hIL-1 β	ATGGACAAGCTGAGGAAGATG	CCCATGTGTCGAAGAAGATAGG
hIL-6	GGAGACTTGCCTGGTGAAA	CTGGCTTGTTCCTCACTACTC
hGapdh	AGCCACATCGCTCAGACAC	GCCCAATACGACCAAATCC
